# Psychiatric Disorders and Substance Use in Homeless Youth: A Preliminary Comparison of San Francisco and Chicago

**DOI:** 10.3390/bs2030186

**Published:** 2012-08-30

**Authors:** Ernika G. Quimby, Jennifer P. Edidin, Zoe Ganim, Erika Gustafson, Scott J. Hunter, Niranjan S. Karnik

**Affiliations:** 1 Pritzker School of Medicine, University of Chicago, 924 East 57th Street, Suite 104, Chicago, IL 60637, USA; E-Mail: egquimby@uchicago.edu; 2 Department of Psychiatry and Behavioral Neuroscience, University of Chicago, 5841 South Maryland Avenue, Chicago, IL 60637, USA; E-Mails: jedidin@uchicago.edu (J.P.E.); elgustafson@uchicago.edu (E.G.); shunter@uchicago.edu (S.J.H.); 3 Department of Education and Early Childhood Development, Government of Australia, Victoria 3001, Australia; E-Mail: zoeganim@gmail.com

**Keywords:** youth homelessness, mental health, substance use

## Abstract

Youth homelessness is a growing problem in the United States. The experience of homelessness appears to have numerous adverse consequences, including psychiatric and substance use disorders. This study compared the frequencies of psychiatric disorders, including substance use, between homeless youth (18–24 years-old) in San Francisco (*N* = 31) and Chicago (*N* = 56). Subjects were administered the Mini International Neuropsychiatric Interview (M.I.N.I.) to assess DSM-IV-TR diagnoses and substance use disorders. Eighty-seven percent of the San Francisco youth, and 81% of the Chicago youth met criteria for at least one M.I.N.I. psychiatric diagnosis. Nearly two-thirds of the youth in both samples met criteria for a mood disorder. Approximately one-third met criteria for an anxiety disorder. Thirty-two percent of the San Francisco sample and 18% of the Chicago met criteria for Antisocial Personality Disorder. Approximately 84% of the San Francisco youth and 48% of the Chicago youth met criteria for a substance-related disorder, and more substances were used by San Francisco youth. In conclusion, the high rate of psychiatric disorders in homeless youth provides clear evidence that the mental health needs of this population are significant. Implications are discussed.

## 1. Introduction

An estimated 1.7 million youth are homeless in the United States [1]. These youth are at risk for a myriad of problems including low academic achievement, psychopathology, and physical health problems. The causality of the relationship among these factors has not been determined [2]. Nevertheless, the effects of homelessness on these youth may be potentially long lasting. Specifically, experiences during adolescence are thought to have an impact on brain development and impaired development can in turn lead to problems including increased risk taking behaviors [3,4,5,6]. Such findings may help explain why homeless youth have substance use rates that are double that of their housed counterparts [7,8]. Rates of numerous psychiatric disorders are higher among homeless youth, including mood, anxiety, and substance use disorders, and it has additionally been found that homeless youth often have co-morbid psychiatric diagnoses [9].

The role of geographical location in experiences and outcomes of youth homelessness is an additional factor that warrants attention, as regional differences have been found across the country among youth, especially in areas such as risk exposure and behaviors [10,11]. Despite this, most services available for homeless youth are relatively standardized nationwide, assuming a homogenous population [12,13]. Attempts to increase the current underutilization of both mental health and substance related services may be furthered by tailoring such services to the varying needs of homeless youth [14,15].

This study specifically investigated frequencies of psychiatric disorders among homeless youth, including substance-related disorders in two cities (Chicago and San Francisco) where data collection was made possible by collaborations between members of our research team and local homeless youth service organizations. We pursued this comparison study in order to test the pragmatics for conducting research between two sites and to examine potential regional differences between homeless populations. Comparisons of the rates of disorders between homeless youth in two cities, Chicago and San Francisco, were made. It was hypothesized that, consistent with prior research, rates of psychiatric disorders and substance use (alcohol and other drugs), would be high among the homeless youth. Regional differences between the two samples were expected. A more thorough understanding of the high rates of substance-related and other psychiatric disorders, including regional differences in presentation, can be used to develop more targeted support and interventions for homeless youth.

## 2. Methods

### 2.1. Participants

San Francisco: Youth were recruited from the Larkin Street Youth Services drop-in center in San Francisco. Members of the research team were on-site in the drop-in center and center staff referred youth to the research team in order to participate in the study. Each subject had to be between the ages of 18 and 25 and speak English. After informed consent was obtained, each participant completed both a semi-structured interview with a trained research assistant and a self report questionnaire.

Chicago: Participants for the study were homeless youth who met the following inclusion criteria: (1) Individuals who “lack a fixed, regular, and adequate nighttime residence” [16] including youth who temporarily share the housing of others due to financial hardship; (2) ≥18 years-old; (3) <25 years old; (4) reside in Chicago; (5) speak English, and (6) have a measured IQ ≥ 55. Participants were recruited from two Chicago agencies, The Night Ministry and Teen Living Program. Staff members at each of the agencies initiated contact with potential participants. Interested youth were contacted by the study coordinator or research assistant, and youth for whom consent was obtained were enrolled in the study.

Demographic information is summarized in Table 1. Research in each city was approved by an institutional review board that was associated with the providers and research sites.

**Table 1:** Participant demographics.

City	San Francisco		Chicago	
	N	%	N	%
**Subjects**	31	100	56	100
**Age**				
Average	20.9	--	19.3	--
(SD)	(1.85)		(0.9)	
Range	18–24	--	18–21	--
**Gender**				
Female	7	22.6	30	53.6
Male	24	77.4	26	45.6
**Ethnicity**				
African American	10	32.2	50	89.3
Caucasian	16	51.6	0	0.0
Latino	3	9.7	0	0.0
Multiracial	0	0.0	3	5.4
Other	2	6.5	3	5.4

### 2.2. Procedure

In both cities, participants were administered the Mini-International Neuropsychiatric Interview (M.I.N.I.) [17]. Version 5.0 was used in San Francisco and 6.0 in Chicago. The M.I.N.I. allows for the assessment of DSM-IV-TR diagnoses and substance use disorders, via a semi-structured interview. It has strong sensitivity and specificity for the majority of disorders [18]. It has high reliability (kappa 0.88–1.0) and validity (kappa usually greater than 0.5). Furthermore, its design allows for rapid exclusion of diagnoses by using pertinent negatives, limiting the time needed to complete an evaluation to about 45–60 min.

### 2.3. Statistical Analyses

Descriptive data were calculated for the frequencies of the various psychiatric disorders, as assessed by the M.I.N.I., in both populations. Chi- square tests were used to determine whether diagnoses of psychiatric and substance use disorders differed between the Chicago and San Francisco samples.

## 3. Results

### 3.1. Sample Characteristics

In San Francisco, 31 homeless youth (mean age 20.9, SD 1.8; 22.6% female; 32.0% African American, 52.0% Caucasian, 10.0% Latino) participated. In Chicago, participants included 56 homeless youth (mean age 19.3, SD 0.9; 53.6% female; 89.3% African American).

### 3.2. Psychiatric Disorders

Eighty-seven percent of the San Francisco youth, and 82% of the Chicago youth met criteria for at least one psychiatric diagnosis on the M.I.N.I. The average number of diagnoses was four in both San Francisco and Chicago. The percentage of youth with any mood, anxiety, or psychotic disorder were approximately 61%, 32%, and 13%, respectively, in San Francisco, and 63%, 36%, and 7%, respectively, in Chicago. Table 2 shows the specific M.I.N.I. diagnoses that were assessed in both cities. Chi square analyses were not significant, with the exception of Hypomanic Episode, Past.

**Table 2:** Mini International Neuropsychiatric Interview (M.I.N.I.) diagnoses.

City	San Francisco		Chicago		Significance of City		
	(*N* = 31)		(*N* = 56)				
Diagnosis	No.	%	No.	%	*χ^2^*	*df*	*p*
Any psychiatric disorder *	27	87.1	46	82.0	12.0	10	ns
Any mood disorder	19	61.3	35	62.5	4.1	4	ns
Any anxiety disorder	10	32.2	20	35.7	2.3	4	ns
Any psychotic disorder	4	12.9	4	7.1	0.6	1	ns
Major Depressive Episode	10	32.3	10	17.9	2.3	1	ns
Major Depressive Episode, Recurrent	10	32.3	12	21.4	1.2	1	ns
Suicidality	18	58.0	35	62.5	0.2	1	ns
Manic Episode	1	3.2	4	7.1	0.6	1	ns
Manic Episode, Past	2	6.5	8	14.3	1.2	1	ns
Hypomanic Episode	1	3.2	0	0.0	1.8	1	ns
Hypomanic Episode, Past	12	38.7	10	17.9	8.0	1	0.007
Panic Disorder	2	6.5	0	0.0	3.7	1	ns
Agoraphobia	3	9.7	10	17.9	1.0	1	ns
Social Phobia	4	12.9	7	12.5	0.0	1	ns
Obsessive Compulsive Disorder	2	6.5	4	7.1	0.0	1	ns
Posttraumatic Stress Disorder	1	3.2	7	12.5	2.2	1	ns
Psychotic Disorder	3	9.7	3	5.4	0.6	1	ns
Mood Disorder, with psychotic features	1	3.2	2	3.6	0.0	1	ns
Generalized Anxiety Disorder	6	19.4	6	10.7	1.2	1	ns
Anti-Social Personality Disorder	10	32.3	10	17.9	2.2	1	ns

* Any positive screen on M.I.N.I.; disorders are current, unless otherwise noted.

### 3.3. Substance Use

The rates of substance-related M.I.N.I. diagnoses, abuse and dependence of alcohol and substances, are shown in Table 3. Nearly 84% of the San Francisco youth and 49% of the Chicago youth met criteria for a M.I.N.I. diagnosed substance-related disorder. Rates of dependence were higher than abuse in San Francisco (45.1% *vs.* 38.7%), but approximately equal in Chicago (25.0% *vs.* 23.2%). Also, in each city, the rate of non-alcohol substance dependence or abuse (58.1% in San Francisco; 28.6% in Chicago) was higher than alcohol dependence or abuse (48.3% in San Francisco; 19.6% in Chicago). Chi square analyses revealed no significant differences in substance abuse disorders across cities. The *p* value for alcohol dependence approached significance (*p* = 0.057).

**Table 3:** M.I.N.I. diagnoses of substance-related disorders.

City	San Francisco		Chicago		Significance of City		
	(*N* = 31)		(*N* = 56)				
Diagnosis	No.	%	No.	%	*χ^2^*	*df*	*p*
Substance Dependence	11	35.5	9	16.1	2.0	1	ns
Substance Abuse	7	22.6	7	12.5	1.5	1	ns
Alcohol Dependence	10	32.2	5	8.9	4.5	1	0.057
Alcohol Abuse	5	16.1	6	10.7	0.5	1	ns
Any Dependence	14	45.1	14	25.0	8.5	2	ns
Any Abuse	12	38.7	13	23.2	12.6	2	ns

Table 4 shows the most frequent non-alcohol substances used by homeless youth who met M.I.N.I. criteria for dependence or abuse. In both groups, cannabis was the main substance used. In San Francisco, an equal number of people reported cocaine, narcotic, and stimulant use as the next most used substances. One Chicago participant reported abuse of cough medicine, while cannabis was the only substance of dependence or abuse among the remaining Chicago youth.

**Table 4:** Most frequent substances used *.

Substance	N	Substance	N
San Francisco		Chicago	
Cannabis	12	Cannabis	15
Cocaine	2		
Narcotics (heroin, methadone)	2		
Stimulants (methamphetamine, amphetamine)	2		
		Cough medication	1

* Among M.I.N.I. Substance Abuse/Dependence; Non-Alcohol.

Youth in San Francisco endorsed a wider range of substances used, in the absence of a M.I.N.I. substance use diagnosis. Over ten specific different substances were reported among the youth in San Francisco, including at least one in the stimulant, cocaine, narcotic, hallucinogen, inhalant, cannabis, and tranquilizer categories on the M.I.N.I. In Chicago, the use of four specific drugs (marijuana, cough medicine, ecstasy, and Xanax) was reported.

## 4. Discussion

As hypothesized, and consistent with prior studies, rates of psychiatric disorders among homeless youth were high across both San Francisco and Chicago. Based on frequency reports alone, several key similarities and differences were noted. These findings also supported our expectations, although few comparative studies currently exist (none comparing Chicago and San Francisco to our knowledge).

Demographically, the San Francisco sample was more ethnically diverse and older. The gender composition of the samples also differed, with relatively more females in the Chicago sample. Youth in San Francisco endorsed a greater number of psychiatric concerns overall; however, with the exception of Hypomanic Episode, Past, these differences did not reach significance. With regard to substance use, abuse, and dependence, no significant differences were noted between San Francisco and Chicago youth, although alcohol dependence approached significance. It is of note that San Francisco youth reported a greater number and range of specific substances used.

There were several limitations to this study. First, this study has relatively small sample sizes that have the potential to skew the sample. It is difficult to draw inferences about the representativeness of the sample to an external population because so little is known about the overall homeless population and also due to the dynamic nature of this population. Second, the type of shelter from which the participants were recruited was not matched in this preliminary study, and features of each could potentially have influenced the participant characteristics identified. Larkin Street Youth Services, the San Francisco recruitment site, is the primary provider of homeless youth services in San Francisco, and draws youth from across the city and thereby might explain the greater ethnic diversity. In contrast, the sites in Chicago drew from more localized areas, in part due to Chicago’s greater degree of racial and ethnic segregation, larger size, and availability of multiple centers. Neither the San Francisco nor Chicago sites drew from youth living on the streets. Looking further into differences in shelter sites and also sampling from the streets could be a next step, since, for example, rates of substance use have been found to be different between those homeless youth who spent more time living on the streets compared to those who lived more consistently in shelters [19]. Other demographic differences were noted between the samples, including the gender and racial/ethnic composition, as well as the average age of youth at the different sites. These differences may affect patterns of psychiatric disorders and substance use. The small sample size prohibited additional analyses to examine these relationships in more detail. The sample size may have precluded finding significant results due to lack of power in the current samples. Future research will address some of these limitations, particularly by having larger sample sizes.

The U.S. Department of Health and Human Service’s Healthy People 2020 highlights the need for further study in such priority topics as “mental health and mental disorders,” and “social determinants of health” [20]. The current study confirms this need, as it is clear that homelessness is a key element in a combination of multiple factors that contribute to unmet health needs. A better understanding of the associations between neuropsychological, psychiatric, and social variables, with attention paid to regional differences in the experiences and outcomes of homelessness, may clarify the relative impact of homelessness on these aspects of functioning. Gathering a greater range of demographic data, such as age of first homeless experience, length of time spent homeless, and education attained (which is currently being collected in our ongoing Chicago research) may further provide a more comprehensive understanding of the unique factors of the experience of homelessness that inform functioning.

Future studies would benefit from longitudinal designs, as they would provide improved information about how psychiatric and substance use disorders begin and develop over time in this population. It would also help clarify the relative impact of homelessness on the development of these disorders. In particular, it would be useful to collect data from younger youth and youth at-risk for homelessness in order to follow them over time. Although difficult, this would likely require that youth be recruited prior to becoming homeless. This could potentially be accomplished through various recruitment strategies such as working with schools, religious organizations, and community agencies to recruit youth who might be at-risk for homelessness. Recruitment of street youth, rather than those in shelters, may also increase the age range of samples. To improve the likelihood that youth will remain in the study, studies may benefit from the utilization of creative methods to maintain contact with study participants. Recent studies have begun to employ social networking technologies (e.g., cell phones, email, and Facebook) to provide information to participants and may be useful in the maintenance of long-term relationships with investigators, as it would improve our understanding of other factors that increase risk of psychiatric and substance use disorders. Finally, future studies may benefit from additional sources of data collection, as self-report may yield inaccurate results.

Ultimately, the findings from future studies would assist researchers in the development of new prevention and intervention programs. Currently, studies suggest that there are few effective academic, medical, and psychological interventions specifically designed for homeless youth [21,22]. A better understanding of the experience of homeless youth and the factors that impact their psychiatric functioning would assist in the development of future interventions so that they are appropriately targeted for this population. This would likely lead to improved utilization rates and effectiveness of the programs. It may behoove investigators to work with assistance from homeless youths themselves and those who work with the population on a regular basis.

## 5. Conclusions

The high rate of undiagnosed psychiatric disorders in homeless youth is an alarming observation, although not surprising, and provides clear evidence that the mental health needs of this population are significant. With the particularly high rates of substance-related disorders observed, the need for appropriate prevention and intervention approaches with this community is strong [23]. The vast majority of adults who suffer from substance abuse began using substances during adolescence, and the effects of use at that age have associations with poorer psychosocial and mental health outcomes during later life [24,25]. Regional data collected in this study highlight important information regarding how best to tailor services to increase utilization and eventual outcomes for homeless youth.
